# Task-related gaze control in human crowd navigation

**DOI:** 10.3758/s13414-019-01952-9

**Published:** 2020-01-28

**Authors:** Roy S. Hessels, Andrea J. van Doorn, Jeroen S. Benjamins, Gijs A. Holleman, Ignace T. C. Hooge

**Affiliations:** 1 Experimental Psychology, Helmholtz Institute, Utrecht University, Heidelberglaan 1, 3584CS, Utrecht, The Netherlands; 2 Developmental Psychology, Utrecht University, Utrecht, The Netherlands; 3 Social, Health and Organisational Psychology, Utrecht University, Utrecht, The Netherlands

**Keywords:** Gaze, Task, Social interaction, Wearable eye tracking, Human crowds, Social affordances

## Abstract

Human crowds provide an interesting case for research on the perception of people. In this study, we investigate how visual information is acquired for (1) navigating human crowds and (2) seeking out social affordances in crowds by studying gaze behavior during human crowd navigation under different task instructions. Observers (*n* = 11) wore head-mounted eye-tracking glasses and walked two rounds through hallways containing walking crowds (*n* = 38) and static objects. For round one, observers were instructed to avoid collisions. For round two, observers furthermore had to indicate with a button press whether oncoming people made eye contact. Task performance (walking speed, absence of collisions) was similar across rounds. Fixation durations indicated that heads, bodies, objects, and walls maintained gaze comparably long. Only crowds in the distance maintained gaze relatively longer. We find no compelling evidence that human bodies and heads hold one’s gaze more than objects while navigating crowds. When eye contact was assessed, heads were fixated more often and for a total longer duration, which came at the cost of looking at bodies. We conclude that gaze behavior in crowd navigation is task-dependent, and that not every fixation is strictly necessary for navigating crowds. When explicitly tasked with seeking out potential social affordances, gaze is modulated as a result. We discuss our findings in the light of current theories and models of gaze behavior. Furthermore, we show that in a head-mounted eye-tracking study, a large degree of experimental control can be maintained while many degrees of freedom on the side of the observer remain.

## Introduction

Human crowds present an interesting case for the study of vision. Navigating human crowds requires locomotion while avoiding obstacles, both those which are fixed to the world and those which move. Researchers have long been interested in how locomotion is controlled by visual information (e.g., Gibson (1958), Patla (1997), and Warren (1998)), and how crowd behavior can emerge from visual control of locomotion of multiple individuals (e.g., Moussaïd et al., (2011), Bonneaud and Warren (2012), and Warren (2018)). Thus, on the one hand, human crowd navigation may be considered as a steering task, where visual information is primarily used to navigate safely without colliding. On the other hand, the objects of interest in crowd navigation are other humans, who are often considered to be special for perception and cognition (e.g., Atkinson et al., (2011)). The faces and bodies of other humans may carry important visual information about potential interactions (i.e., whether one could strike up a conversation, or whether one might need to prepare for aggressive behavior). In this regard, it has been shown that human faces, for example, tend to attract and maintain attention (e.g., Bindemann et al., (2005) and Langton et al., (2008)), as evidenced by longer reaction times to competing objects in the presence of faces. In human crowd navigation, visual information may therefore be acquired for multiple processes, i.e., the navigation itself and seeking out social affordances. In this paper, we investigate how visual information is acquired for these two processes by studying gaze behavior during human crowd navigation under different task instructions.

A ubiquitous factor in visual control is the eye-movement system. When a saccade is made, the part of the world that is within the visual field may change dramatically, and with it the locations in the world that are projected onto the high-resolution fovea and lower-resolution peripheral areas of the retina. Whether foveal scrutiny is required, or occurs, for a given task depends on the nature of that task. Graybiel et al., (1955), for example, reported that figure skaters and slalom runners’ performance dramatically drops when peripheral vision is reduced, not when central vision is reduced. Similarly, Owens and Tyrell (1999) reported that steering accuracy in a driving task is disrupted by reduction of the visual field, but not by severe blur and luminance reduction. Based on these studies, one might expect that foveal scrutiny is not required primarily for the task of steering through a human crowd. Although the high resolution of central vision is not always required or sufficient for task performance, gaze location is often tightly linked to the task being carried out: e.g., while steering a car (Land & Lee, 1994), making tea (Land et al., 1999), or while making sandwiches (Hayhoe, 2000). The studies of Land and Lee (1994), Land and Furneaux (1997), Land et al., (1999), and Hayhoe (2000) have led to the conclusion that gaze behavior is functionally relevant for many behaviors and often precedes the behavior which gaze subserves. For example, gaze location precedes the action of grabbing a kettle or a tea cup when making tea. In what follows, we review previous work on (1) the allocation of gaze during locomotion and human crowd navigation and (2) the role of gaze in seeking out social affordances. Finally, we discuss potential interactions of these two processes and introduce our present study.

### The allocation of gaze during locomotion and human crowd navigation

Although foveal scrutiny may not necessarily be required for effective steering behavior, humans may adopt certain gaze strategies during locomotion and human crowd navigation. Previous eye-tracking research on the allocation of gaze during locomotion has, for example, shown that the eyes and head lead the way when changing direction during locomotion (Hollands et al., 2002) and that looking at a crossing pedestrian predicts passing behind that pedestrian (Croft and Panchuk, 2018). Further eye-tracking work has shown that one tends to look away from the direction of gaze of an oncoming virtual pedestrian and skirt the pedestrian on that side (Nummenmaa et al., 2009). These studies indicate that gaze is an important predictor of where one might navigate. However, few studies have been conducted using eye tracking to investigate how gaze may support navigation through actual crowds. One reason for this may be, as Berton et al., (2018) point out, that “such studies can be difficult to organize in real crowds because of technical, human, and experimental organization” (p. 1). Instead, researchers have used virtual reality (e.g., Jovancevic et al., (2006), Nummenmaa et al., (2009), and Berton et al., (2018)), or a limited number of people (e.g., Jovancevic-Misic and Hayhoe (2009) and Croft and Panchuk (2018)) to investigate gaze during e.g., pedestrian avoidance.

In a relevant virtual reality study, Jovancevic et al., (2006) had participants complete two tasks: Either to avoid virtual pedestrians or to avoid virtual pedestrians and follow a leader walking in front of them. The virtual pedestrians were programmed such that some would change their path towards the participant for 1 s. The authors reported that the number of fixations to those ‘colliding’ pedestrians was higher than for non-‘colliding’ pedestrians, but only when the task was to avoid pedestrians, not when also to follow a leader. The fact that ‘colliding’ pedestrians were looked at more often was deemed surprising by the authors, given that a pedestrians never actually collided with a participant: “We conjecture that obstacle/pedestrian avoidance is a highly learned activity that may be difficult to extinguish within an hour of testing. Collisions with obstacles and pedestrians in real life are quite rare. Presumably, there is some avoidance system engaged to prevent such collisions even when the frequency of actual collisions is rather low” (Jovancevic et al., 2006, p. 1446). In a follow-up study with actual people, Jovancevic-Misic and Hayhoe (2009) reported that participants fixated potential ‘colliders’ more often and did so proactively only after a few encounters. In more recent work, Meerhoff et al., (2018) recorded gaze direction while participants had to move by means of a joystick through a virtual environment containing multiple walking agents. They found that gaze was often directed to agents that had a high risk of colliding and were subsequently avoided. The authors argue that “humans navigate through crowds by selecting only few interactions and that gaze reveals how a walker prioritizes these interactions” (p. 248). In sum, these studies show that gaze tends to be allocated to pedestrians that are on a collision course with the observer, and that gaze direction can predict where one might walk.

While the studies described above answer where gaze is allocated during locomotion and human crowd navigation, they do not reveal when gaze needs to be allocated to a particular region of the visual world in order to complete a task. A recent set of studies has been conducted on this topic from the perspective of outside lighting conditions (e.g., Davoudian and Raynham (2012) and Fotios et al., (2015c)). The main question these studies address is what the lighting conditions at night should be, such that pedestrians’ critical visual tasks can be successfully completed. In a series of studies (Fotios et al., 2015a, ??b, ??c, 2018), participants walked through different residential areas while wearing eye-tracking glasses. Fotios et al., (2015a) had participants respond to auditory tones randomly presented throughout their walk. When response time to such a tone was relatively long (more than twice the standard deviation above the mean), participants were assumed to be involved in a ‘critical visual task’. During these instances, participants looked more at other pedestrians and the path than at any other area, which is interpreted as other pedestrians and the path being task-relevant gaze locations while navigating in residential areas. Fotios et al., (2015b) stated that fixations to other pedestrians are expected, given that their behavior is less predictable than that of objects or goals. As an alternative explanation for why pedestrians are fixated, Fotios et al., (2015b) state that fixating pedestrians may be required in order to accurately perceive their motion and speed, although no evidence is presented to show that this is the case. What is particularly interesting is that Fotios et al., (2015b) stated that “Regarding fixations on people, the human tendency for social attention means there is a bias towards fixation on other people when they appear in a scene and this may be regardless of their apparent movement or behavior” (p. 157-158). This brings us to the second process that may be relevant in human crowd navigation, namely seeking out social affordances.

### Gaze and social affordances

Human faces carry important information for potential interactions, for example identity, emotion, and gaze direction, which is relevant information when deciding to engage in or refrain from interaction. A person’s gaze direction, for example, can hold valuable information regarding that person’s spatial locus of attention (Langton et al., 2000), a fact that is often exploited by magicians (e.g., Tatler et al., (2007) and Ekroll et al., (2017)). In other words, faces convey important social affordances. Previous research has consistently shown that human faces tend to attract and maintain attention (e.g., Bindemann et al., (2005) and Langton et al., (2008)), although see Pereira et al., (2019) for a contrasting viewpoint. Bindemann et al., (2007), for example, have shown that faces attract attention automatically, whereas objects do not. This may be overcome, however, by voluntary control when objects are more likely to cue the upcoming location of a target. In other words: faces attract attention automatically in the presence of objects, yet when objects are task-relevant and faces are not, this information can be used to locate targets faster. Another consistent finding described in the literature is that humans, human faces, and/or eyes are preferentially looked at (Frank et al., 2012; Birmingham et al., 2009; Van der Geest et al., 2002; Johnson et al., 1991; Pelphrey et al., 2002; Walker-Smith et al., 1977; Henderson et al., 2005), although it depends on what information is needed at a particular moment in time (e.g., Võ et al., (2012)). The fact that human faces and eyes tend to attract and maintain attention and gaze is colloquially referred to by phrases or questions such as “the eyes have it” or “do the eyes have it?” (Langton et al., 2000; Emery, 2000; Võ et al., 2012; Pereira et al., 2019). Based on this literature, one might construct a *special-human hypothesis*, which holds that the human form attracts and holds one’s gaze regardless of “their apparent movement or behavior” (Fotios et al., 2015b, p. 157-158), whenever the task doesn’t explicitly require other locations in the world to be looked at (e.g., Bindemann et al., (2007)).

Recent research suggests that a more nuanced version of this *special-human hypothesis* is appropriate, namely that gaze to people is also context-dependent. Laidlaw et al., (2011), for example, showed that a confederate was fixated when visible through a video feed in a waiting room, but not when that confederate was physically present. Gobel et al., (2015) showed participants videos of people low and high in social rank and told the participants that these people would either see them as well, or not. The eyes of people with a high social rank were fixated less when participants were told that the other would see their video compared to when they would not. In a study particularly relevant to ours, Foulsham et al., (2011) showed that people tend to look less at others at a near distance when they walk around campus compared to when they watch a video of someone walking around campus. On the basis of the work by Laidlaw et al., (2011), Gobel et al., (2015) and Foulsham et al., (2011), it has been concluded that gaze in social settings (e.g., when other people are present) not only serves visual information uptake but may also signal information to others and is therefore dependent on the social context (see also Jarick and Kingstone (2015) and Risko et al., (2016)). We wonder how these findings apply to human crowd navigation, where visual control for avoiding collisions and seeking out potential social affordances presumably co-occur. Tentative evidence that social affordances are automatically sought out in human crowds comes form work by Gallup et al., (2012b), who showed that people shift their gaze direction based on where people in their neighborhood look. Further work by the same group has revealed that such shifts in gaze direction depend on e.g., the emotion portrayed by someone, and the walking direction relative to the observer (Gallup et al., 2012a; Gallup et al., 2014).

### The present study

In the present study, we investigated the allocation of gaze during human crowd navigation. Our first question was where and for how long gaze is allocated to human bodies, faces, objects, and the environment when navigating through crowds. To answer this question, observers wore eye-tracking glasses and walked through hallways containing crowds and objects while instructed to avoid collisions. We recruited a large group of volunteers to participate as crowds. We thus extend previous work in virtual reality by Jovancevic et al., (2006) and work with a small number of pedestrians by Jovancevic-Misic and Hayhoe (2009). By using a fully scripted scenario, we aimed to produce a similar visual stimulus for all our participants wearing eye-tracking glasses. In outdoor wearable eye-tracking studies (e.g., Fotios et al., 2015a, ??b, ??c, 2018), this is not feasible. Second, we investigated how gaze during human crowd navigation depends on whether social affordances have to be explicitly sought out, by adding a second task. The second task was to assess whether oncoming people make eye contact or not, which is a relevant task for everyday life with potential social consequences. Eye contact is, for example, important when finding a person in a crowd, when deciding when to pass someone or not (Croft & Panchuk, 2018), or during conversation (Argyle, 1972; Ho et al., 2015; Hessels et al., 2019). By using dual tasks, we can (1) investigate whether gaze shifts and fixations are the bottleneck for safely navigating human crowds. If they are, posing a dual task may lead participants to slow down, bump into others, or show longer fixation times. If gaze shifts and fixations are not the bottleneck for safely navigating human crowds, we can (2) investigate the flexibility with which gaze can be directed to other people for seeking out social affordances.

## Methods

### Participants

Participants were recruited at the Faculty of Social and Behavioral Sciences of Utrecht University. A total of 49 people volunteered. Participants were assigned one of two roles; either to walk through the lab center with eye-tracking glasses on (henceforth referred to as *observers*) or to walk through the lab center in the direction opposite to the observer’s walking direction (henceforth referred to as *walkers*). Eleven observers (six male, five female, mean age = 22.55 years, range, 20–27 years) and 38 walkers (17 male, 21 female, mean age = 24.21 years, range, 20–38 years) participated in the study. The final number of participants was determined as follows. First, a large group had to be amassed at the same time to conduct the study. We estimated that we were able to gather around 50 participants at the same time. Second, walkers had to walk two rounds for each observer, meaning that we could not have an unlimited number of observers as this would severely strain the walkers (in total, the walkers walked an estimated 4 km). As such, we estimated a running time of 1 h for the experiment itself, and 1 h for instructing and training the walkers. At an estimated 5-6 min required per observer, we could test 11 observers within an hour. The remaining participants (38 in this case) acted as walkers. Most of the participants came from the same Bachelor and/or Master program at our Faculty, which meant that observers would encounter familiar walkers during the experiments. We did not deem this to be a problem, as walking around any campus for any student likely involves meeting acquaintances along the route.

Written informed consent was obtained from all participants prior to the start of the study. Participants were compensated with 2.5 so-called ’participant hours’, of which Psychology students have to acquire 12 during their studies, if they were eligible. The study was approved by the Ethics Committee of the Faculty of Social and Behavioral Sciences at Utrecht University (protocol number FETC18-075) and adhered to the Declaration of Helsinki.

### Apparatus

Eye movements of the 11 observers were measured using the Tobii Pro Glasses 2 (firmware version 1.25.3-citronkola). Eye movements were recorded at 50 Hz, while the scene camera recorded at 25 Hz. Recordings were made using the Tobii Pro Glasses Controller (version 1.95.14258), running on an HP Pavilion X2 with Windows 10. In order to record manual responses from the observers, a one-button device was built, which was connected to the Tobii Pro Glasses recording unit using a 3.5-mm jack plug. Pressing the button yielded a 3.3-V signal into the Sync-in Port on the recording unit, which was recorded straight into the eye-tracking data.

### Setup lab center

**Fig. 1:**
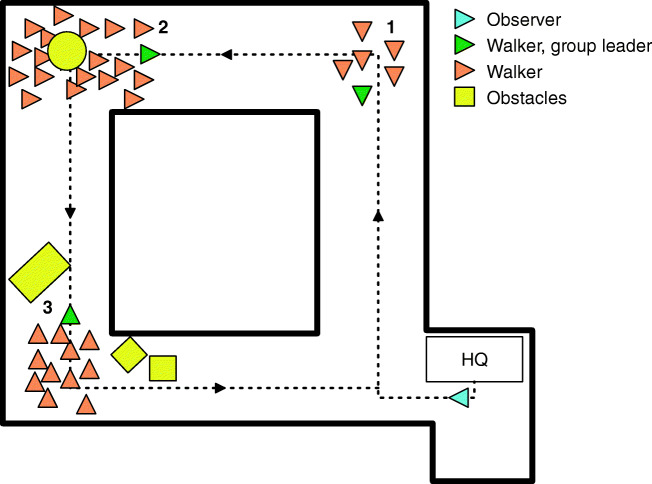
Schematic overview of the lab center with the starting positions of the three groups of walkers (*orange arrowheads*, with group leaders in *green arrowheads*) and observer (*light blue arrowhead*). *Arrowheads* point into the walking direction. The *black arrows* indicate the route of the observer. Headquarters (HQ) indicate where the eye-tracking glasses were fitted. Obstacles are marked in *yellow*. Each group of walkers is characterized by its number. The corridors were 40-45 m in length, and roughly 2.25 m wide

The Psychology lab center at Utrecht University consists of four corridors at 90° turns (see Fig. 1). The corridors are approximately 40 to 45 m long and 2.25 m wide. Each group of walkers (see *Procedure*) was positioned at one corner of the lab center. The observer and walkers walked in opposite directions.

**Fig. 2:**
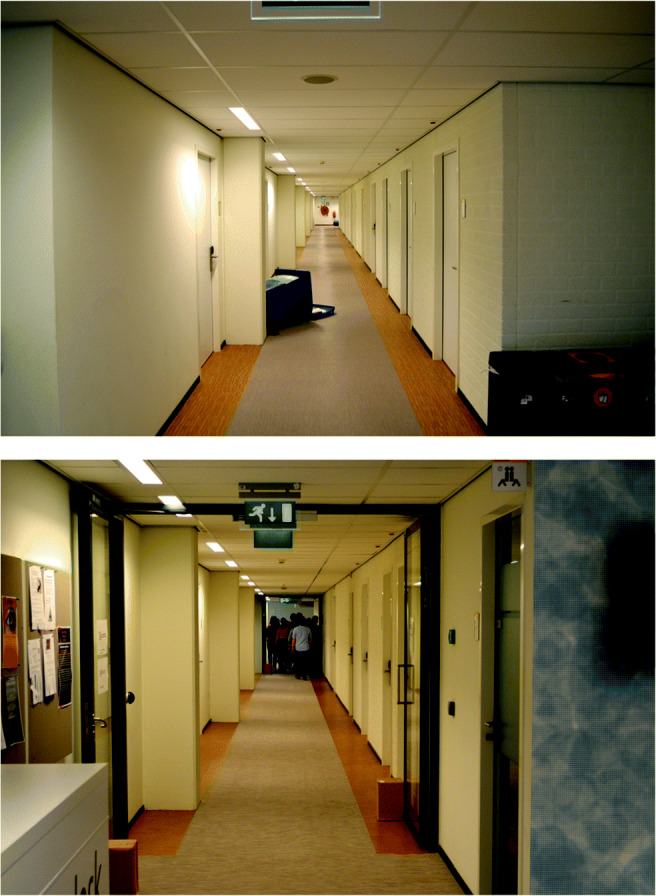
Two corridors of the lab center in which the present study was conducted. The *top panel* depicts the tipped trashcan as described in *Setup lab center*, photographed from the starting position of group 3 (see Fig. 1). The *bottom panel* depicts the largest group of walkers in the background, photographed from the corner closest to the observer starting position (see Fig. 1). The photograph is taken from the back to protect the privacy of the walkers

At several locations in the lab center, obstacles were placed. These obstacles, marked in yellow in Fig. 1, consisted of a round seat, a tipped trashcan, and two upright trashcans. The round seat was 0.8 m in diameter and positioned with its center at 1 m from the wall. The area in which it was positioned was slightly wider than the rest of the corridor, leaving 0.6 m on one side and 2 m on the other side for the participants to pass. The tipped trashcan was positioned such that it took up roughly half the width of the hallway, leaving about 1-1.2 m of walking space. The two upright trashcans (0.6 m wide, 0.7 m deep, 1.05 m high) were placed just around a corner and created a funnel so that roughly 1.4 m was left for the participants to walk through. At two locations, doors that normally close off the lab center were held open with small cardboard boxes. Figure 2 depicts two of the corridors of the lab center.

Note that we initially piloted the experiment in larger, less constrained, spaces. However, conducting a crowd-navigation experiment in such large spaces requires many more participants than we could amass. Furthermore, the data analysis for such large spaces proved less feasible, particularly with regard to mapping fixations to the world.

### Procedure

Upon arrival, written informed consent was obtained from the 49 volunteers. Each volunteer was subsequently assigned the role of either observer or walker. Walkers immediately received an individualized instruction sheet (full instructions are available at https://osf.io/4upbn/) with how they were to walk through the lab center. Observers were sent with author JB to a separate room for instruction.

The individualized instructions for the walkers comprised the following information. First, walkers were assigned into one of three groups. For each group, one group leader was assigned who was given a walkie-talkie (Motorola TLKR-T80 Extreme Quad). This group leader was to start walking together with their group when instructed through the walkie-talkie by the experimenters, and to stop once they had reached their starting position in the lab center. No walker was allowed to pass the group leader. To prevent that walkers would walk neatly on one side of the corridor and would all look away from the observer, a subset of the walkers in each group was given additional instructions. Roughly 30% of the walkers were instructed to look at the observer when (s)he passed in the corridor (henceforth called *watchers*). Another 15–20% of the walkers were instructed to cross in front of the observer as (s)he passed in the corridor (henceforth called *obstructors*). Finally, groups were instructed to walk on the left or the right side of the corridor, or to take up 80% of the corridor width. A breakdown of walker instructions in each group is given in Table 1. When all walkers had understood their instructions, the groups were each positioned in one corner of the lab center, and a practice round was conducted.

**Table 1:** Breakdown of walker instructions in each group

Group	Number of walkers	Group leader	Watchers	Obstructors	Walking location
1	6	1	2	1	Left side of corridor
2	20	1	7	4	80% of corridor width
3	12	1	4	2	Right side of corridor

Observers were instructed that they were to walk rounds through the lab center wearing eye-tracking glasses, but that detailed instructions were to be given just prior to the start of their round. One by one, observers were fitted with the eye-tracking glasses by one of the experimenters (author JB), after which the eye-tracking glasses were calibrated using the one-point calibration marker provided by Tobii Pro held at arm’s length by the observer. After calibration, observers were escorted a short distance where a display with a 3 by 3 grid of validation markers was positioned on the wall. Each validation marker consisted of a red dot of 0.9 cm in diameter surrounded by a black ring 4.5 cm in diameter. The distance between observer and the wall was 65 cm. The center validation point was at 155 cm height. These values are given in centimeters, not degrees, as we did not have exact measures of eye height for all participants. Assuming an eye height of 170 cm and a distance between observer and wall of 65 cm, the distance between the cyclopean eye and the center validation marker (155 cm height) was 66.71 cm. Given this distance, the 0.9-cm inner red circle and the 4.5-cm black ring correspond to 0.77° and 3.86°, respectively. Distance between the three rows of markers was 10 cm and the distance between the three columns of markers was 16 cm. Observers were asked to fixate each point for at least 1 s. After the validation, observers were escorted to a second experimenter (author RH).

The second experimenter instructed the observer that (s)he had to walk one round through the lab center and make sure not to bump into anything or anyone. The button that was attached to the Tobii Pro Glasses recording unit was to be ignored for now. After verifying that the observer had understood the instruction, the experimenter then called into the walkie-talkie for the groups to start walking, and sent off the observer. After returning to the experimenter, instructions for a second round were given. Observers again had to walk a round through the lab center and make sure not to bump into anything or anyone. This time, however, observers were asked to press the button whenever they thought that someone was making eye contact with them. After having given the new instructions, the experimenter called into the walkie-talkie for the groups to start walking and sent off the observer. At the conclusion of the second round, the first experimenter escorted the observer to the nine-point validation display, where a second validation sequence was conducted. Finally, the eye-tracking glasses were removed from the observer and the next observer was prepared. The duration of one measurement from first to second validation took between 5 and 7 min. Each individual round took roughly 1.5 to 2 min.

Note that the order of the instructions was not counterbalanced across participants. We decided not to do this after careful consideration. We expected that if we would have asked half our participants to first assess eye contact and avoid collisions, they might still be focused on assessing eye contact in the round when they only have to avoid collisions. In other words, we expected that if we would have chosen to use this order of instruction, gaze behavior in the second round may be dependent on the instruction in the first round. On the contrary, we did not expect gaze behavior in the second round to be dependent on the instructions in the first round for the order of instructions that we used (first avoiding collisions, then avoiding collisions *and* assessing eye contact). Navigating through crowds and avoiding collisions is a daily task for many people, and the observers in our experiment are also familiar with the lab center. As such, we do not see how observers might be better ‘trained’ in the second round than in the first round in navigating crowds and avoiding collisions. As such, we deemed the order of instructions of first avoiding collisions, and then avoiding collisions and assessing eye contact, to be the most sensitive method for picking up task-related difference in gaze behavior.

After all 11 observers had walked two rounds through the lab center, all volunteers were given an exit interview. This interview comprised demographic questions (age, gender, handedness), a question about whether their vision was normal or corrected-to-normal, and what they thought this study was about and whether they noticed anything in particular. The entire experiment took around 3 h to complete, including the informed consent procedure, the training of the walkers, conducting all measurements and debriefing the participants.

### Data analysis

#### Video coding

The videos that were recorded from the scene camera of the Tobii Pro Glasses 2 were analyzed to determine when the observers’ rounds began and ended, and when observers passed groups. The videos were coded using Datavyu 1.3.7 (Datavyu Team, 2014) by authors JB and AD. The following six types of events were coded:

1. Beginning of a round: first frame in which first hallway door is completely visible
2. Group comes into view
3. First person of a group is out of view
4. Last person of a group is out of view
5. Obstructer is out of view
6. End of a round: experiment leader starts talking to observer

After a first round of coding, a number of missing Datavyu codes were identified, which were added by author JB.

#### Eye-tracking data analysis

##### Eye-tracking data quality

The quality of the eye-tracking data was assessed by three characteristics: (1) the accuracy, or systematic error in the eye-tracking data, (2) the precision, or variable error in the eye-tracking data, and (3) data loss. The systematic error was assessed manually by looking at the gaze replays of the validation at recording start and end in GlassesViewer (Niehorster et al., 2020). GlassesViewer is open-source software that can be used to view raw eye-tracking data and videos from the Tobii Pro Glasses 2. It is available from https://github.com/dcnieho/TobiiGlassesViewer/. The variable error was estimated by the median root mean square sample-to-sample deviation (RMS). The RMS deviation was computed for each recording using GlassesViewer. A 300-ms moving window was slid over the azimuth and elevation signals of the left and right eye separately, and for each signal the median RMS deviation was calculated. By taking the medians, the velocities during fast changes in gaze direction (e.g., saccades) are excluded from the measure. Data loss was estimated as the percentage of samples without a gaze coordinate. Data loss can occur due to e.g., blinks, but also due to technical problems in tracking the eyes by the eye tracker (see e.g., Hessels et al., (2015)).

##### Fixation classification

In this study, we take the definition of fixation that Hessels et al., (2018b) used in their wearable eye-tracking example: “A fixation is a period of time during which an area of the visual stimulus is looked at and thereby projected to a relatively constant location on the retina” (p. 21). Fixations were operationalized by a slow-phase classifier based on Hooge and Camps (2013). In Hooge and Camps (2013), slow phases are classified based on an adaptive velocity threshold. Initial application of that classifier to our eye-tracking data revealed that fast phases were often missed during periods of overall low gaze velocity, and too many fast phases were classified in periods when the overall gaze velocity was high. We therefore modified the classifier such that the adaptive velocity threshold depended on a time window of 8 s, instead of the entire recording (see Appendix ?? A for additional figures demonstrating the difference in fixation classification between the two methods).

Specifically, slow-phase classification proceeded as follows. First, the gaze velocity signal was estimated for each sample by the displacement to the previous and next sample divided by the time differences. These two values were subsequently averaged. Then, a moving window of 8-s length was slid over the data. For each window, all velocities higher than the average plus 2.5 standard deviations were removed iteratively until the velocity threshold converged to a constant value or the number of iterations reached 200. The velocity threshold for that window was then determined as the average velocity plus three standard deviations. The final velocity threshold for each sample was the average of the thresholds in the windows it had been part of. All samples below the velocity threshold were labeled as potential fixation samples. Fixations were operationalized as sequences of potential fixation samples equal to or larger than 80 ms.

##### From fixations to area of interest measures

In order to determine where observers looked throughout the measurement, fixations needed to be mapped to the world (people, objects, walls, etc.). Given that many computer-vision techniques exist for finding humans in videos, we attempted to use such techniques to make our area of interest (AOI) analysis objective and reproducible (as we have done for face-viewing studies before, Hessels et al., (2018a)). However, all the techniques that we tried (e.g., OpenPose) would not adequately detect humans further than a few meters away from the observer. This was likely partly due to motion blur and the wide-angle image of the Tobii Pro Glasses 2 scene video. Therefore, fixations were manually mapped unto AOIs. This was done using GazeCode (Benjamins et al., 2018). GazeCode is an open source tool for manually mapping fixations unto AOIs. Manual mapping in GazeCode has been proven to be faster than in the Tobii Pro software. Coding categories can easily be tweaked, and there is full control over which fixation classifier is used. Raw gaze data and videos with overlaid raw gaze data were exported from Tobii Pro Lab (version 1.76.9338) and imported into GazeCode. Three coders mapped the fixations unto AOIs: authors RH, GH, and IH. Fixations were assigned to one of seven AOIs:

1. Objects on the ground—including the objects positioned by us, as well as naturally occurring objects such as trashcans and sofas against the wall.
2. Walls—including objects on the wall such as fire hoses and posters or signage.
3. Group in the distance—meaning gaze is on one or more persons walking towards the observer, but it cannot be distinguished on which person, and where on that person.
4. Body walker
5. Head walker
6. Body obstructor—see section on *Inter-rater reliability* (below) on problems in distinguishing obstructors from walkers.
7. Head obstructor

When a fixation was not on any of the categories above, it was assigned to the Non-AOI, which thus included the floors and the end of a corridor. Henceforth, fixation duration refers to the median of an observer’s fixation durations and total fixation duration to the sum of all fixation durations. If not specified, they refer to fixations regardless of the AOI they were assigned to. As the AOIs could occur at different distances from the observer, it is important to note that the Tobii Glasses 2 features automatic parallax compensation.

Fixations were only analyzed if they began after, and ended before, the start and end times of the rounds as coded by both video coders.

## Results

This section is structured as follows. First, the inter-rater reliability, eye-tracking data quality and task performance are described. Hereafter, the AOI-based eye-tracking measures are described. Sufficient inter-rater reliability and eye-tracking data quality are required to make sense of the eye-tracking data and the relation between fixation location and the visual stimulus. Task performance is assessed, as we want to know whether any changes in gaze behavior across rounds are related to task performance or not.

### Inter-rater reliability

The inter-rater reliability of the manual mapping of fixations to AOIs was assessed by computing Cohen’s kappa for all possible pairs of coders. Cohen’s kappa was between 0.58 and 0.74. Confusion matrices revealed that there was disagreement between the obstructor categories and the walker categories. As gaze to the obstructors was not our main interest, codes for the ‘Head obstructor’ category were changed to the ‘Head walker’ category, and codes for the ‘Body obstructor’ category were changed to the ‘Body walker’ category. For this reduced AOI set, Cohen’s kappa was between 0.66 and 0.76, which qualifies as ‘substantial’ agreement according to Landis and Koch (1977). It further compares favorably to Fotios et al., (2018), who report a Cohen’s kappa of 0.54 for the classification of ‘head’, ‘body’, and ‘unknown’ categories.

From the codings of the three coders, one final set was generated. If two or more coders agreed on a category for a fixation, that category was used. If three coders disagreed, the codes of one coder were used (author RH). This occurred for 4% of all fixations. As a sensitivity analysis, we reran all analyses with the codings of each individual coder (e.g., author RH, GH, IH). The conclusions reported below are not dependent on whether we use the codings of one coder or the other.

### Eye-tracking data quality

The systematic error in the eye-tracking data was assessed manually for the validation at recording start and end by author RH. If the systematic error is higher at recording end than at recording start, this can indicate that something occurred during the recording which leads the eye-tracking data to be unreliable for interpretation. Gaze was mostly on or close to the inner red circle of the validation marker (0.9 cm in diameter or roughly 0.77°) and well within the black ring (4.5 cm in diameter or roughly 3.86°) for all nine validation points. Furthermore, no appreciable differences between the offset between gaze and validation marker were observed from recording start to recording end. The means and ranges for the other two measures of eye-tracking data quality: the variable error (RMS deviation) and data loss (percentage samples without a gaze coordinate) are given in Table 2. Note that the RMS deviation values are substantially higher than when commonly reported as a measure for variable error (e.g., Hessels et al., (2017)). These values are usually in the 0-0.3° range when reported for world-fixed eye trackers (e.g., remote or tower-mounted eye trackers) and immobile participants. In our case, however, RMS deviation is computed throughout a measurement in which a participant is walking. This means that the eyes are almost always moving with respect to the head. These RMS values do, however, allow between-participant comparisons of data quality. Based on the observed values, an assessment of all the gaze replays in GlassesViewer and an assessment of the fixation classifications, we decided that eye-tracking data quality was sufficient for our purposes and therefore did not exclude any participant.

**Table 2:** Eye-tracking data quality—means and ranges for the variable error and data loss

Signal	RMS deviation (°)	Data loss (%)
Left eye azimuth	0.81 (0.51-1.48)	9.07 (4.57-17.76)
Left eye elevation	0.70 (0.36-1.64)	9.07 (4.57-17.76)
Right eye azimuth	0.78 (0.54-1.40)	7.82 (3.27-14.57)
Right eye elevation	0.71 (0.33-1.82)	7.82 (3.27-14.57)

### Task performance

Two tasks were posed to the observers. In their first round, observers had to navigate the hallways while avoiding collisions. In their second round, observers furthermore had to assess eye contact made by oncoming walkers. Performance on the first task is defined by (1) the number of collisions, and (2) the time taken to walk a round. Performance on the second task (assessing eye contact) could not be quantified, as we did not know the number of walkers that looked at the participant. Although 13 *watchers* were instructed to look at the observer, other walkers may have done so as well. However, we can assess whether observers carried out the task at all, by determining the number of times the button was pressed to indicate eye contact. Note also that we refer to ‘single task’ and ‘dual task’ below, which only signifies the fact that the second round contained an additional task compared to the first round. Of course, ‘navigate a hallway and avoid collisions’ can be considered to consist of many sub tasks. However, it is the difference in gaze between the two ‘tasks’ that matters.

**Fig. 3:**
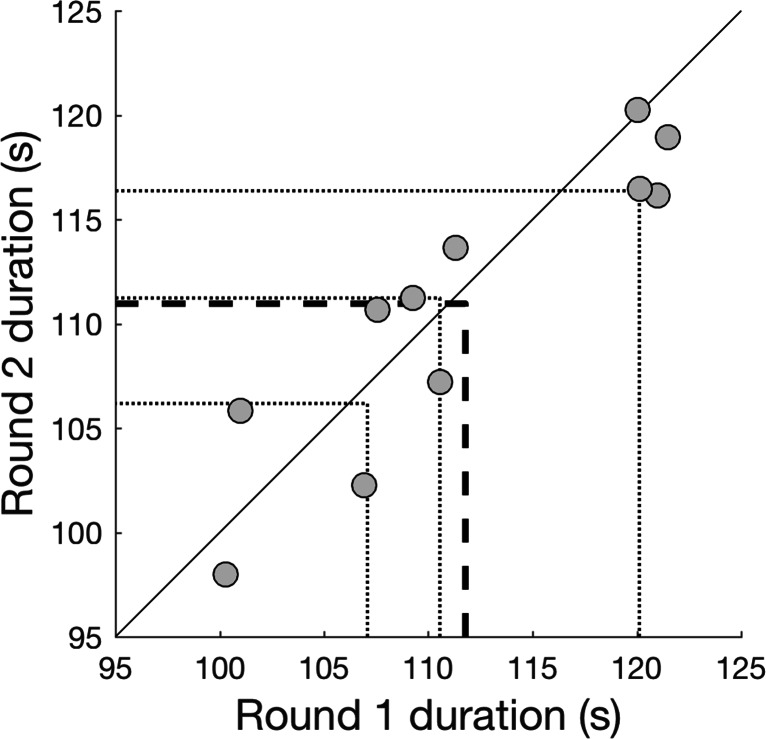
Duration of rounds 1 and 2 as a performance measure of hallway navigation while avoiding collisions. Each round took between 95 and 125 s, i.e., roughly 1.5 to 2 min. Round duration is defined as the time between the start of the first fixation and the end of the last fixation that fall completely within the round start and end times as coded by both video coders. Each *dot* represents one observer. The *black line* corresponds to unity. *Dotted lines* indicate the 25th, 50th (median), and 75th percentile. The *bold dashed line* indicates the mean

As can be seen from Fig. 3, observers’ time in rounds 1 and 2 are close to the unity line. This indicates that observers took roughly the same time to complete each round. This was confirmed by an intraclass correlation coefficient for absolute agreement of 0.90 (ICC(A,1), see Weir (2005) and McGraw and Wong (1996)). Furthermore, no collisions were observed for any observer. As such, performance for navigating hallways while avoiding collisions was unaffected by the addition of the dual task (assessing eye contact). Furthermore, all observers carried out the assessment of eye contact, as indicated by the fact that all observers identified multiple instances of eye contact (mean number of button presses = 35, range, 24-46).

### Area Of interest-based measures of gaze

#### What attracts or maintains gaze during human crowd navigation?

**Fig. 4:**
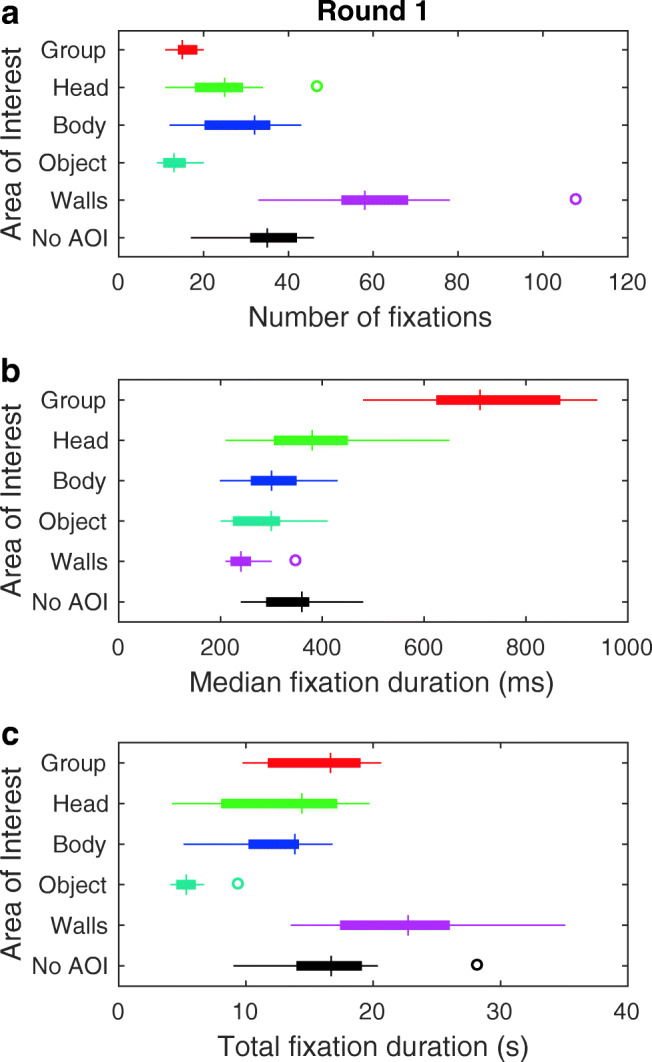
Measures of observers’ gaze behavior in round 1. *Panels* depict box and whisker plots for **a** the number of fixations **b** median fixation duration, and **c** total fixation duration. Box and whisker plots are organized by area of interest. Medians are indicated by the *vertical bars*. *Boxes* cover the 25th to 75th percentiles (inter-quartile range; IQR). *Whiskers* extend from the 25th and 75th percentile to cover all participant data lying within 1.5 times the IQR from the 25th and 75th percentile, respectively. Any participant data lying outside this range are identified by an *open circle*. The ‘No AOI’ encompasses all fixations not on any of the other AOIs (e.g., to the floor)

We first investigated what attracts and maintains gaze during human crowd navigation. As a measure for gaze attraction, we used the number of fixations to the different AOIs (bodies, heads, objects, walls, etc.). As a measure for gaze maintenance, we used the median fixation duration to the different AOIs.^1^ Figure 4 depicts these measures of observers’ gaze behavior for round 1. We restrict ourselves to the analysis of round 1, as here the only task for the observers was to avoid collisions. We present the analysis of gaze behavior in round 2 when we discuss task-dependence of gaze during human crowd navigation below. As can be seen from panel A, observers looked most often at the walls, followed by the body and head of the walkers. The group at a distance and objects were looked at least often. Note that although the number of fixations is indicative for what attracts gaze, it is also determined to a large extent by the availability of walls, people and objects over time. I.e., if fewer people and more objects are placed in the lab center, the number of fixations to these AOIs is likely to change. An interesting comparison in this regard is heads versus bodies, as they belong to the same entity and are thus not affected by the availability: A head often occurs in the presence of an accompanying body, although a body may be hidden behind another walker for some time. As visible from panel A in Fig. 4, observers seem to have looked about equally often at the heads and bodies of oncoming walkers.

As visible from panel B in Fig. 4, groups at a distance maintained gaze substantially longer than the other AOIs. Walls tended to maintain gaze for the shortest median duration. Although groups at a distance maintained gaze longer than other AOIs, it should be noted that it is the only AOI for which the distance between observer and the object of the AOI (the group in this case) could not be smaller than at least several meters. If the distance between the group and observer was small (i.e., it could be distinguished on which person and where on the person the observer fixated), fixations were coded as being to the body or head of individual walkers. It may therefore be that fixations to groups at a distance comprised multiple shorter fixations to different people and parts of the body that could not be picked up with our eye tracker.

If human bodies or faces maintain gaze more than objects, median fixation duration is expected to be longer for the body and/or head than for objects. In order to quantify whether bodies and heads maintained gaze longer than objects, two Bayesian paired-samples *t* tests were conducted in JASP (JASP Team, 2018) with the hypotheses that (1) the median fixation duration for heads was longer than the median fixation duration for objects, and (2) the median fixation duration for bodies was longer than the median fixation duration for objects. For all subsequent Bayesian *t* tests, we report both the Bayes factor and the 95% credible interval for the Cohen’s *d* measure of effect size as reported by JASP. Neither hypothesis was supported by the data, as evidenced by Bayes factors of 1.99 for heads (median Cohen’s d = 0.48, 95% credible interval of Cohen’s *d*: [0.05, 1.08]) and 0.39 for bodies (median Cohen’s *d* = 0.22, 95% credible interval of Cohen’s *d*: [0.01, 0.67]) versus objects, respectively. Note that neither the null hypothesis was supported, i.e., that median fixation durations were *not* longer for heads and bodies than for objects. It is furthermore important to note that the fixation duration measure is not affected by the division between heads and bodies, as it is the average duration of each classified fixation. I.e., if we were to consider heads and bodies together as a ‘human’ AOI (excluding the group at a distance, when we cannot distinguish what person is being looked at), the conclusion would not be affected.

**Fig. 5:**
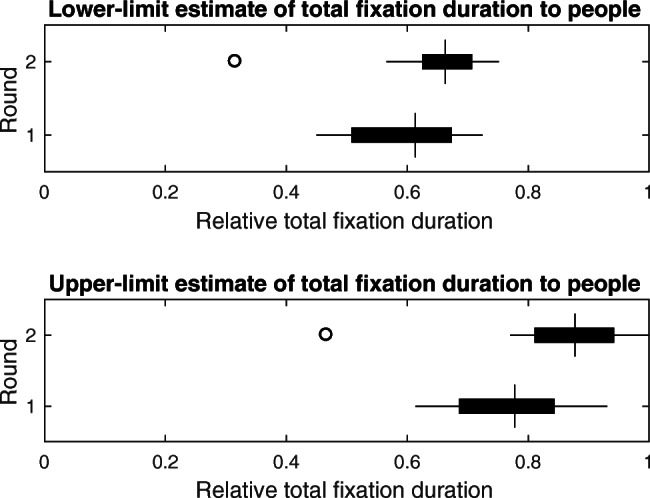
Measures of observers’ gaze behavior to people in rounds 1 and 2. *Box and whisker plots* indicate the relative total fixation duration to people (sum of fixations to the group, head and body) for each round. Medians are indicated by the *vertical bars*. *Boxes* cover the 25th to 75th percentiles (inter-quartile range; IQR). *Whiskers* extend from the 25th and 75th percentile to cover all participant data lying within 1.5 times the IQR from the 25th and 75th percentile, respectively. Any participant data lying outside this range is identified by an *open circle*. Relative total fixation duration is estimated according to two methods. The *top panel* depicts the lower-limit estimate of relative total fixation duration to people, which was calculated by dividing the sum of total fixation durations to the group, body, and head AOIs by the total time that people were in view in the scene camera video. The latter was determined as the time between a group entering the scene camera video and the last person of a group leaving the scene camera video. This is considered a lower limit estimate, as a relative total fixation duration of 1 can only be obtained by one continuous fixation on a group for the entire duration it is in view. However, the eye-tracking data also contains fast phases (saccades) and/or data loss. As such, another estimate was also calculated. The *bottom panel* depicts the upper-limit estimate of relative total fixation duration to people, calculated by dividing the total fixation durations to the group, body, and head AOIs by an estimated maximum time people could be looked at. This estimated maximum time was calculated by first determining the proportion of eye-tracking data that contained fixations, compared to fast phases and/or data loss for the entire eye-tracking recording. This proportion was then multiplied by the total time people were in view in the scene camera video. If for example people are in view for 50 s, and the proportion of eye-tracking data that contained fixations was 0.8, the estimated maximum time people could be looked at was 40 s

As an aggregated measure of gaze attraction and maintenance, total fixation duration for the different AOIs is depicted in panel C of Fig. 4. Overall, gaze was on walls for the longest total duration, followed by the group at a distance, heads and bodies. This raises the question of whether people were always looked at whenever they were in view. In order to answer this question, we summed the total fixation duration to the group, head and body AOIs. This gives us an estimate of the total time observers looked at people. We then estimated the proportion of time that people were looked at when they could have been. This was done in two ways. First, we divided the total fixation duration to people, by the total time that groups were in view in the scene camera video (defined as the time between a group entering the scene camera video and the last person of a group leaving the scene camera video). Note that in all instances the time when a group came into view occurred when they navigated around a corner or appeared from behind another group. In other words, observer orientation in the hallway never occluded a group that might have been visible from the scene camera. This gives us a lower-limit estimate of the proportion of time people are looked at. The reason that this is a lower limit is that the eye-tracking does not only contain fixations (i.e., slow phases, see *fixation classification*), but also fast phases and/or data loss. As such, we calculated a second estimate. This was done by calculating the proportion of time in a round that contained fixations. This was then multiplied by the total time that groups were in view in the scene camera video in order to derive an estimated maximum time people could have been looked at. The total time people were looked at was then divided by the estimated maximum time people could be looked at. This latter estimate of the proportion of time people are looked at can be considered an upper limit. If, for example, people were in view for 50 s, and the proportion of eye-tracking data that contained fixations was 0.8, the estimated maximum time people could be looked at was 40 s. Both estimates are given in Fig. 5. As can be seen, the relative total fixation duration to people in round 1 ranged between 0.45 and 0.72 (lower limit) and 0.61 and 0.93 (upper limit). People were thus not always looked at when they were in view.

#### How is gaze during human crowd navigation task-dependent?

Our second research question was how gaze during human crowd navigation is task-dependent. We investigated this question by giving our observers one or two tasks. As noted above, performance on the first task (avoiding collisions while navigating through crowds and hallways) was not affected by the addition of the second task (assessing eye contact). This facilitates the interpretation of any difference in gaze behavior between the single task and the dual task. We first give a description of the gaze behavior as it occurred in the second round, before we statistically compare gaze behavior between rounds and test the hypothesis that gaze is task-dependent in human crowd navigation.

**Fig. 6:**
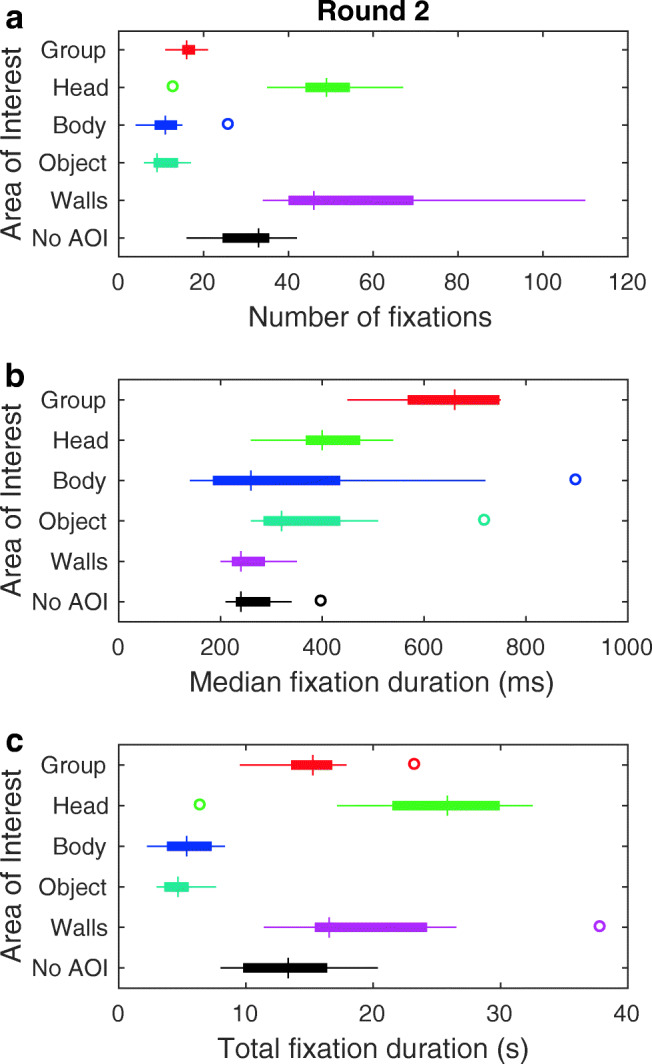
Measures of observers’ gaze behavior in round 2. Panels depict box and whisker plots for **a** the number of fixations, **b** median fixation duration, and **c** total fixation duration. *Box and whisker plots* are organized by area of interest. Medians are indicated by the *vertical bars*. *Boxes* cover the 25th to 75th percentiles (inter-quartile range; IQR). *Whiskers* extend from the 25th and 75th percentile to cover all participant data lying within 1.5 times the IQR from the 25th and 75th percentile, respectively. Any participant data lying outside this range is identified by an *open circle*. The ‘No AOI’ encompasses all fixations not on any of the other AOIs (e.g., to the floor)

Figure 6 depicts number of fixations, median fixation duration and total fixation duration to the different AOIs in round 2. As can be seen from panel A, observers looked mostly at the walls as in round 1, yet this time followed by the head of the walkers. The group at a distance, bodies and objects were looked at least often. As visible from panel B, groups at a distance again maintained gaze substantially longer than the other AOIs. Walls and the ‘No AOI’ (e.g., floors or looking straight into the hallway) tended to maintain gaze for the shortest median duration. Estimates for the relative total fixation duration to people in round 2 (see Fig. 5) were larger than in round 1, indicating that people were looked at more often when in view in round 2 than in round 1. For many observers, this was still not all the time that people were in view.

**Fig. 7:**
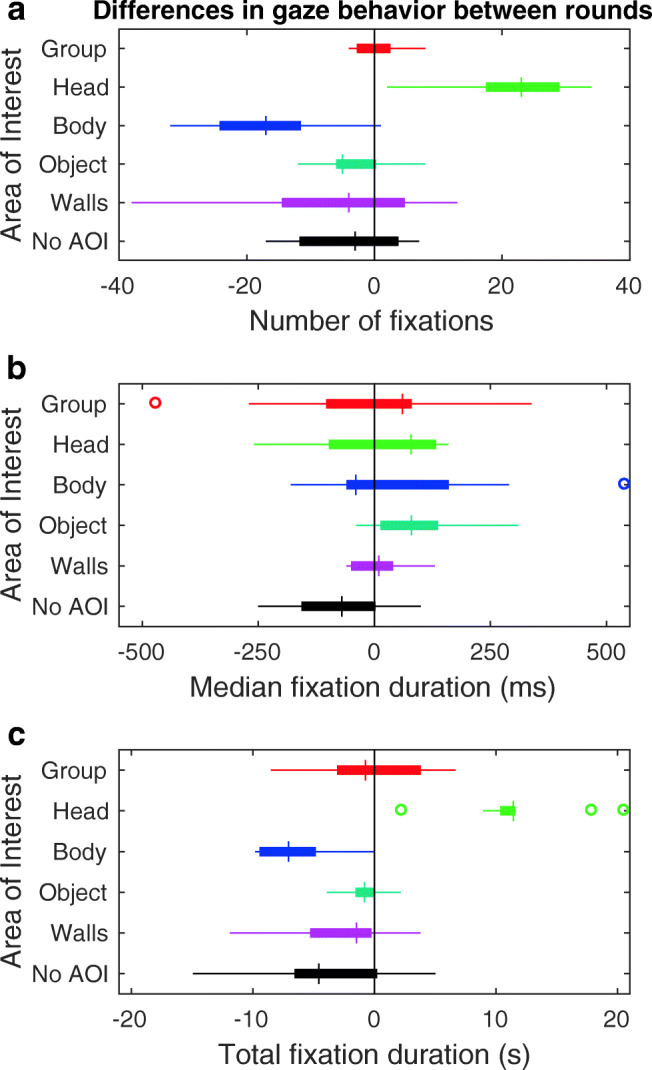
Difference in measures of observers’ gaze behavior between rounds. Panels depict box and whisker plots for the per-participant difference between rounds 1 and 2 for **a** the number of fixations, **b** median fixation duration, and **c** total fixation duration. Differences are calculated by subtracting the value obtained in round 1 (collision avoidance only) from the value obtained in round 2 (dual task collision avoidance and eye-contact assessment). This means that positive numbers indicate increases in round 2 with respect to round 1. Negative numbers indicate decreases in round 2 with respect to round 1. Box and whisker plots are organized by area of interest. Medians are indicated by the *vertical bars*. *Boxes* cover the 25th to 75th percentiles (inter-quartile range; IQR). *Whiskers* extend from the 25th and 75th percentile to cover all participants lying within 1.5 times the IQR from the 25th and 75th percentile, respectively. The *black vertical line* indicates the no-difference point between round 1 and 2. The ‘No AOI’ encompasses all fixations not on any of the other AOIs (e.g., to the floor)

Differences in the measures of gaze behavior between round 1 (single task of avoiding collisions) and round 2 (dual task of avoiding collisions and assessing eye contact) are depicted in Fig. 7. Positive values indicate that the number of fixations, median fixation duration or total fixation duration was higher in round 2 than in round 1. As visible from panel A, the number of fixations in round 2 was higher for the head, and lower for the body, than in round 1. The median fixation durations did not seem to differ across rounds (panel B). Total fixation duration was higher for the head and lower for the body in round 2 than in round 1. These findings were further quantified statistically. Bayesian paired-samples *t* tests were conducted for the number of fixations, median fixation duration, and total fixation duration for each AOI in round 1 versus round 2. The null hypothesis was that there is no difference across rounds. The alternative hypothesis that there were differences across rounds was non-directional, as we had no prior expectation about what the task-dependent modulation of gaze behavior might look like. We only considered Bayes factors larger than 10 or smaller than 0.1, labeled as “strong” evidence in favor of the alternative or null hypothesis in JASP (JASP Team, 2018).

The data supported the hypothesis that the number of fixations to the head in round 2 was different from the number of fixations to the head in round 1 (BF_10_ = 1923, median Cohen’s *d* = - 3.19, 95% credible interval of Cohen’s *d*: [- 4.44, - 2.01]). Similarly, the number of fixations to the body in round 2 was different from the number of fixations to the body in round 1 (BF_10_ = 260, median Cohen’s *d* = 2.36, 95% credible interval of Cohen’s *d*: [1.27, 3.62]). The data furthermore supported the hypothesis that the total fixation duration to the head in round 2 was different from the total fixation duration to the head in round 1 (BF_10_ = 2207, median Cohen’s *d* = - 3.26, 95% credible interval of Cohen’s *d*: [- 4.63, - 1.84]) and that the total fixation duration to the body in round 2 was different from the total fixation duration to the body in round 1 (BF_10_ = 814, median Cohen’s *d* = 2.82, 95% credible interval of Cohen’s *d*: [1.41, 4.17]). No other differences in measures of gaze behavior between rounds 1 and 2 were observed. In sum, this means that observers looked more often at heads and for a longer total duration when avoiding collisions *and* assessing eye contact than when only avoiding collisions. The increase in looking at the heads came at the cost of looking at bodies: observers looked less often at bodies and for a shorter total duration when avoiding collisions *and* assessing eye contact than when only avoiding collisions. Gaze behavior during human crowd navigation was thus task-dependent.

#### Is gaze during human crowd navigation dependent on crowd size?

We investigated gaze during human crowd navigation further by looking at whether gaze to people (bodies and heads) was dependent on the size of the group encountered. We therefore calculated the number of fixations and median fixation duration to the body and head when an observer was walking in a group. Walking in a group was defined as the time between the first group member exiting the scene video of the eye tracker and the last group member exiting the scene video of the eye tracker. As noted before, observers encountered three groups of size 6, 12, and 20, respectively. Each group was encountered twice in a round, which meant that each group was encountered four times in the experiment (i.e., two rounds). The four encounters were collapsed before number of fixations and median fixation duration were calculated.

**Fig. 8:**
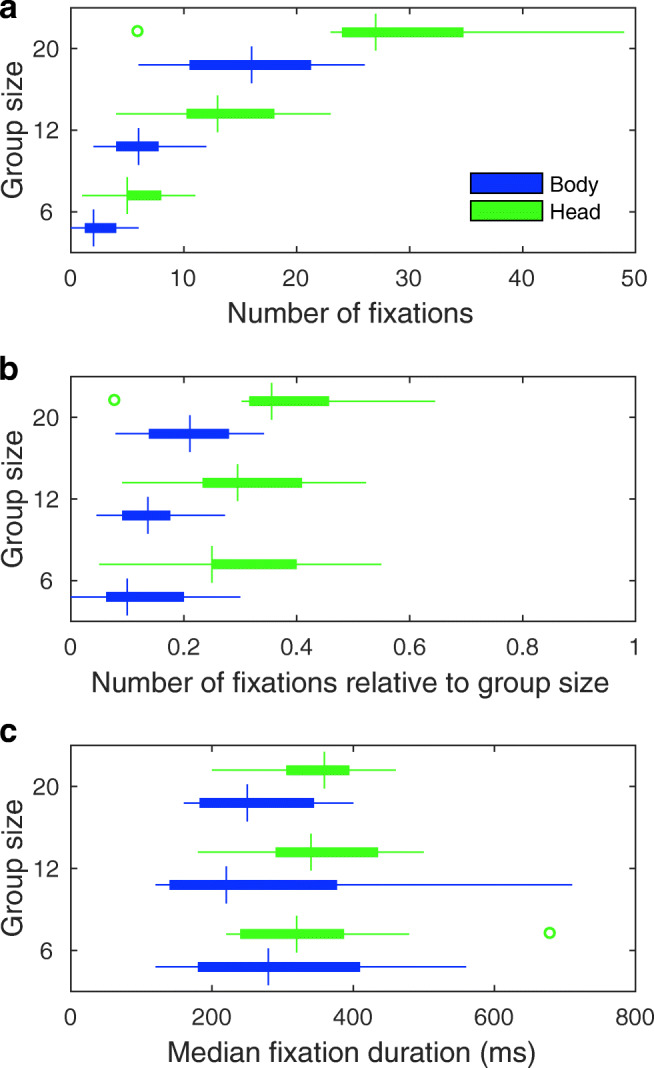
Measures of observers’ gaze behavior to bodies and heads as a function of group size. Panels depict box and whisker plots for the **a** number of fixations, **b** number of fixations relative to group size, and **c** median fixation duration to the body and head as a function of group size. Medians are indicated by the *vertical bars*. *Boxes* cover the 25th to 75th percentiles (inter-quartile range; IQR). *Whiskers* extend from the 25th and 75th percentile to cover all participant data lying within 1.5 times the IQR from the 25th and 75th percentile, respectively. Any participant data lying outside this range is identified by an *open circle*

Panel A in Fig. 8 depicts the number of fixations to the heads and bodies of walkers as a function of group size. As can be seen, the number of fixations to heads and bodies scales with the size of the group encountered. This is to be expected: when there are more people in a group, it is likely that more people are looked at. As we wondered whether larger group sizes meant that relatively more or less people of the group were looked at, we converted the number of fixations to a relative measure. This was done using Eq. 1:

1 $$ \frac{n}{(g-1)*4}  $$

where *n* is the number of fixations that occurred within a group, *g* is the group size, and 4 refers to the number of encounters of a group in our experiment. We divided by the group size minus one, as the first person of the group is already out of view, by virtue of our definition of being ‘in’ a group. A fixation cannot therefore be on that first person of the group. Panel B in Fig. 8 depicts the number of fixations relative to the group size for the body and head AOI. As can be seen, the relative number of fixations appears to increase as a function of group size, albeit only slightly. Bayesian repeated-measures ANOVAs were conducted for the relative number of fixations as a function of group size in JASP (JASP Team, 2018) for the body and head separately (the comparison of gaze to heads versus bodies has already been made above).^2^ These analyses revealed that there was only ‘anecdotal’ evidence (as so-labeled in JASP) in favor of the hypothesis that the relative number of fixations differed across group size for the body AOI, but not for the head AOI (Bayes factors of 3.29 and 0.61 for the body and head models, respectively). It should also be noted that more than one fixation could occur on the same person and that group 3 (with a group size of 12 walkers) was not always fully encountered at the end of a round. As such we are hesitant to claim that the proportion of people looked at in a group differs as a function of group size.

Finally, panel C in Fig. 8 depicts the median fixation duration to the head and body AOI as a function of group size. As can be seen, median fixation duration doesn’t seem to depend on group size for both the body and head AOI. This was confirmed by Bayesian repeated-measures ANOVAs with Bayes factors for the null-model of 3.24 and 4.81 for the body and head, respectively, albeit only ‘anecdotally’. In sum, we find no conclusive evidence that gaze to people within a group depends on the size of that group.

## Discussion

We investigated (1) where and for how long gaze is allocated to human bodies, faces, objects, and the environment when navigating through crowds and (2) how gaze during human crowd navigation depends on whether social affordances have to be explicitly sought out, by posing observers with a dual task of avoiding collisions *and* assessing whether oncoming people make eye contact or not. We hereby investigate whether gaze shifts and fixations are the bottleneck for safely navigating human crowds, as well as the flexibility with which gaze can be directed to other people for seeking out social affordances while navigating crowds. Regarding the latter, the literature on task-control of eye movements would predict that the task predicts gaze locations in the world (e.g., Hayhoe and Ballard (2014)). If one’s task changes, one’s gaze behavior should also change to reflect what the task-relevant goals are. On the other hand, the social attention literature has shown that human faces are special in the sense that they tend to attract and maintain attention (e.g., Bindemann et al., (2005, 2007); Langton et al., 2008)) and are preferentially looked at (Frank et al., 2012; Birmingham et al., 2009; Van der Geest et al., 2002; Johnson et al., 1991; Pelphrey et al., 2002; Walker-Smith et al., 1977; Henderson et al., 2005; Võ et al., 2012). If human faces are always looked at when possible during navigating crowds, making them relatively more important through instructions should not affect gaze behavior. This latter hypothesis was termed the *special-human hypothesis*.

We find no evidence that human bodies or heads maintain gaze longer than objects. Rather, groups at a distance maintained gaze longer than anything else. However, this might have been due to the fact that by definition, a group is always at a distance: it is separated into bodies and heads when the observer comes close. When looking at something from a distance, there is perhaps less need to change gaze direction than when passing an oncoming person in a group. Here, maintaining gaze on that person would eventually require that the observer turns their eyes and/or head substantially to maintain fixation. We also find no evidence that looking at human bodies or heads is dependent on the group size being navigated through. One potential criticism here may be that if differences in e.g., fixation duration on objects versus people are small, a group of 11 observers may not be sufficient to pick up these differences statistically. Indeed, numerically, it seemed that heads maintained gaze longer than objects, both in round 1 and 2. Yet, each observer makes numerous fixations on heads, bodies and objects, and we could therefore also investigate this difference at the individual level rather than the group level. No different picture emerges here. Independent-samples *t* tests on fixation duration for objects versus bodies and objects versus heads (with a liberal alpha of 0.05) indicate a significant difference for only three observers. For one observer, fixation duration to the head is longer than to objects, for another observer fixation duration to the body is longer than to objects, and for still another observer, fixation durations to both the head and body are longer than to objects. All in all, we find no compelling evidence that human bodies or heads holds one’s gaze more than objects while navigating crowds.

Regarding task-dependent gaze control in human crowd navigation, we find that posing observers with an additional task of assessing eye contact has profound effects on the gaze behavior without deteriorating performance on the initial task. Observers were almost as quick to complete a round when performing the single task as when conducting the dual task. In neither case did collisions occur. When the additional task of assessing eye contact was posed, heads were more often looked at than when avoiding collisions alone. This came at the cost of looking at the bodies. It may seem obvious that heads are looked at more when observers were given two tasks, because for one of the tasks the task-relevant location is the heads of oncoming pedestrians. An implication of this finding is that when the task is simply to avoid collisions while navigating crowds, human faces do not always attract and maintain gaze when no other location in the world must be looked at in order to avoid collisions. We thus conclude that gaze during human crowd navigation is task-dependent, and that faces do not attract and maintain one’s gaze whenever there is no immediate task-relevant location in the world to fixate. Note, however, that we did not counterbalance the order of the instructions, for reasons explained in the Methods section. We cannot therefore completely exclude the possibility that the differences in gaze behavior between rounds might be partly due to order effects.

One particularly interesting finding is that differences in gaze behavior between observers were generally small compared to the differences in gaze behavior across rounds. For example, all observers looked more often at the heads in round 2 than in round 1, and all but one observer looked less often at the body in round 2 than in round 1. This matches with conclusions from previous research on human locomotion and navigation. Jovancevic-Misic and Hayhoe (2009), for example, wrote that “What is of interest here is the quantitative aspects of performance. Not only were fixation probabilities very similar between subjects, but so, too, were learning rates, fixation durations, and fixation latencies, despite the complexity of walking in a real, dynamic environment and the lack of explicit instructions.” (p. 6237). It thus seems that the environment, more than the personal characteristics of the observer, determines gaze behavior. On this distinction, Knorr et al., (2016) wrote that:

“interpersonal coordination for successful collision avoidance in human locomotion is not mainly governed by personal characteristics, including physical properties such as height or gender, or personality traits such as aggression or harm avoidance, but rather by the characteristics of the situation such as the properties of the environment, and most importantly the relative positions, speed and heading of the pedestrians.” (p.1342).^3^

**Fig. 9:**
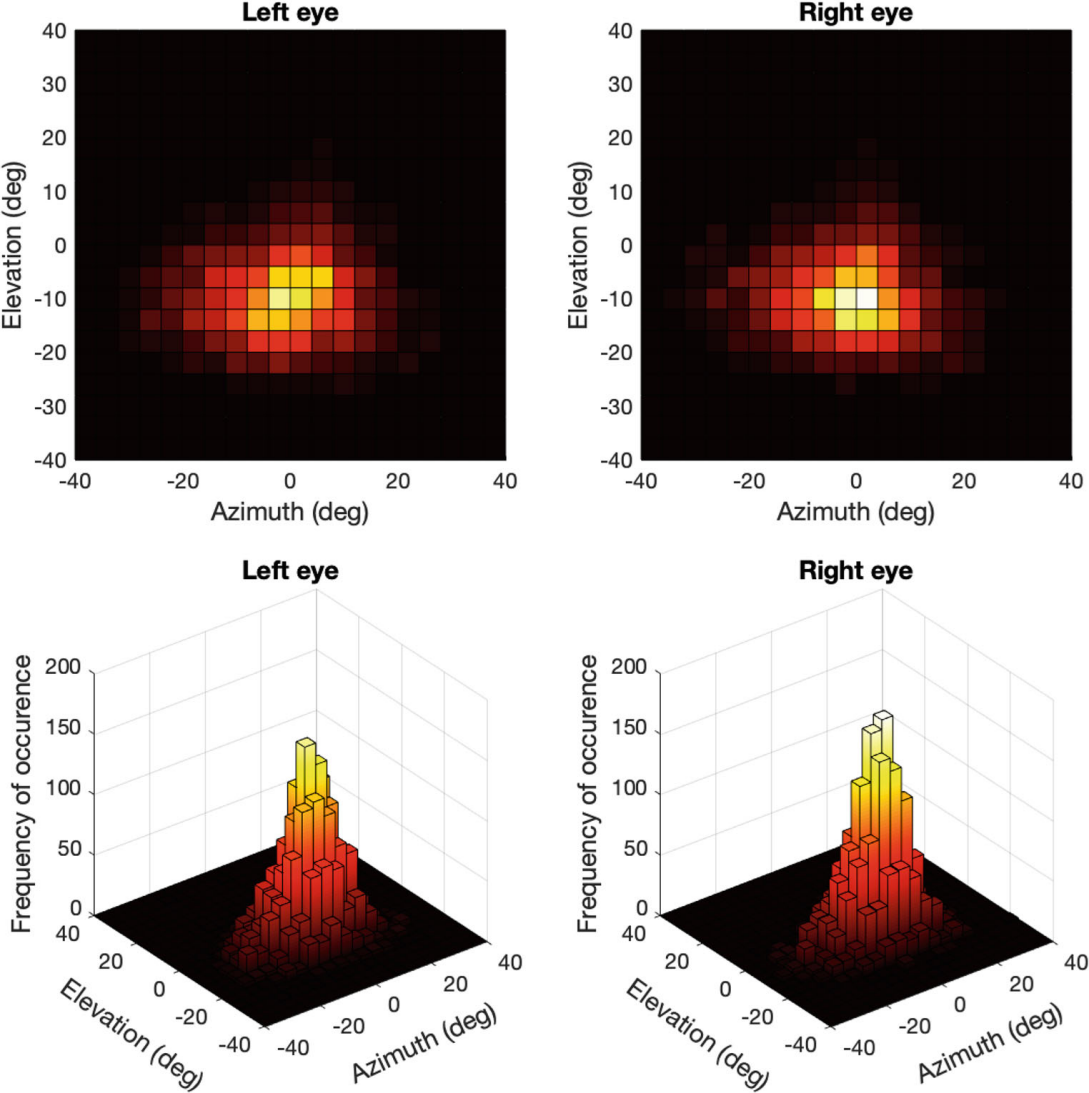
Gaze direction in azimuth and elevation components (in Fick coordinates, see Haslwanter (1995)) with respect to the center of the scene camera of the Tobii Pro Glasses 2 (see https://www.tobiipro.com/product-listing/tobii-pro-glasses-2-sdk/). The *left panels* depict azimuth and elevation for the left eye. *Right panels* depict azimuth and elevation for the right eye. *Top panels* represent the 2D-histogram of gaze directions during fixations, where brighter colors indicate a higher frequency of occurrence. *Bottom panels* depict the same histograms, but depicted as a 3D view. All fixations from all participants were pooled for this figure. Per-participant analyses of gaze direction yield the same pattern; only the maximum of the histogram is shifted slightly per participant

One question that our study raises, is how the observed gaze behavior may be compared to that predicted by theories or models. Is, for example, looking at heads for 15 s in a 90-s walk more or less than expected? This is a difficult question to answer, as psychological theories or models hardly provide point predictions. In our present study, we can only conclude that people are not always looked at whenever they are in view. However, in remote eye-tracking studies with pictures or videos, it is not uncommon to compare human gaze behavior to gaze behavior predicted on the basis of stimulus features (i.e., saliency models, see Itti and Koch (2000) and Itti and Baldi (2005)) or image-independent biases (i.e., the saccadic flow model, see Clarke et al., (2017)). How do such studies compare to ours? For one, in these studies the experimenter chooses what the observer is presented with (pictures or videos) and how it is presented: generally within the frame of a computer screen. In head-mounted eye-tracking studies such as ours, however, the observer moves around and thereby ‘frames’ the world him/herself. What is in the visual field at any point in time, is not only determined by what the experimenter decides to present (e.g., a lab center with crowds and objects), but also by the body, head, and eye movements of the observer. Furthermore, directing one’s line of sight to a target in the world is for the most part solved by rotations of the chest and head, not the eyes (Radau et al., 1994). To demonstrate that this is also the case in our study, we determined the direction of all fixations that occurred during our experiment. These are depicted in Fig. 9. As can be seen, most fixations fell within - 10 to 10 ° for the azimuth component (left eye mean: - 1.65°, *sd* = 10.27°, right eye mean: - 1.50°, *sd* = 10.00°), and between 0 and -20 ° for the elevation component (left eye mean: - 9.41°, *sd* = 7.24 °, right eye mean: - 9.40°, *sd* = 6.89°), indicating a slightly downward gaze direction with respect to the center of the scene camera. Note that the recorded azimuth values ranged from - 42 to 49° and the elevation values from - 38 to 36°, which means that it is unlikely the tracking range of the eye tracker is a limiting factor here.

How might then, a saliency model be applied to a study such as ours, where participants wear a head-mounted eye tracker and navigate through the world? One could argue that the video of the eye-tracker scene camera could be used, as it records the world from the perspective of the observer. Yet, the video of the eye-tracker scene camera is only a depiction of what might have been visible to the observer over time, not what the observer has been ‘presented’ with. The field of view of the scene camera video is smaller than that of the observer—82° horizontal by 52° vertical for the scene camera, and more than 160° horizontal and 70° vertical for the observer with the eye tracker on^4^—and the resolution and contrast of the scene camera video are lower than those of the visual world. Furthermore, the video is already the result of whatever the observer decides to direct their head towards. Applying a saliency model to such a video to investigate how salience may predict gaze therefore seems illogical. More so because salience is often defined on the basis of certain image features, whereas our observers move through a visual world, not a collection of images. What is the spatial scale at which one would represent the visual world in this case? If the scene camera video is taken as a representation of what the observer might have seen, it is a heavily filtered version of the visual world at best. A central-bias model likewise seems illogical to apply to our current study. What would be the frame of reference in which to model a central bias? Is this with respect to the head (or the scene camera video)? This doesn’t necessarily correspond to the center of the observer’s visual field. Or should, in this case, the bias be modeled with respect to the center of the hallway? As mobile eye-tracking studies are increasingly common in psychological research, we believe these are important questions to consider.

The implications of our work are at least threefold. First, our findings lend credence to the idea that the visual control for navigation may operate separately from seeking out social affordances through visual scrutiny. Second, we show that the theoretical framework of task-control of eye movements (Sprague and Ballard, 2004; Sprague et al., 2007; Hayhoe & Ballard, 2014) may apply to settings where other people are involved (i.e., social settings). While we are not the first to use ‘people’ as the predominant task-related objects, which Jovancevic et al., (2006) and Jovancevic-Misic and Hayhoe (2009) have done before, we are, to the best of our knowledge, the first to explicitly distinguish the task-control and social-attention perspectives. This is particularly relevant, given previous claims in the literature about why humans are looked at. As stated before, Fotios et al., (2015b) claim that “Regarding fixations on people, the human tendency for social attention means there is a bias towards fixation on other people when they appear in a scene and this may be regardless of their apparent movement or behavior” (p. 157–158). Clearly, humans are considered to be preferentially fixated. Yet, we find no evidence that humans are particularly special in maintaining gaze compared to other task-relevant locations: e.g., objects that similarly need to be avoided. There is thus a need to integrate both the task-control and social-attention perspectives to understand how gaze supports ongoing behavior across social and non-social settings.

Third, our work is important in the context of recent research on the context-dependence of gaze to humans, and faces in particular. In contrast to research showing that pictures and videos of humans and human faces are preferentially fixated, recent studies have shown that in certain contexts, real humans might not be fixated, for example in a waiting room (Laidlaw et al., 2011). One explanation for this is the fact that the norm in a waiting room is not to interact and thus humans are not fixated, while such norms don’t apply to pictures or videos. Clearly, a theory of gaze, or more generally vision, should be able to explain and predict gaze across contexts. Our findings, together with recent work on the role of social context and task demands in dyadic (face-to-face) communication (Macdonald and Tatler, 2018; Hessels et al., 2019), provide the necessary empirical foundation for such an integrated theory.

A final important outcome of our study is that experimental designs such as ours are feasible. Previous work on gaze while navigating the world, or avoiding pedestrians has been limited to virtual reality (Jovancevic et al., 2006), using only a small number of pedestrians (Jovancevic-Misic & Hayhoe, 2009), or measuring gaze behavior outside with little experimental control (e.g., Fotios et al., (2015a, ??b, ??c, 2018)). We recruited a large number of volunteers that constituted our crowds, and used a fully scripted scenario with the aim of producing a similar scenario for all our observers. We found that the maximum difference in the duration of a round between participants was around 20% of the longest round. Furthermore, differences between subsequent rounds of the same observer were much smaller. As such, we believe that our study presents a good example of how experimental control can be maintained, while many degrees of freedom on the side of the observer remain. We hope that future studies extend our study to large, less constrained spaces.

## Figures and Tables

**Fig. 10:**
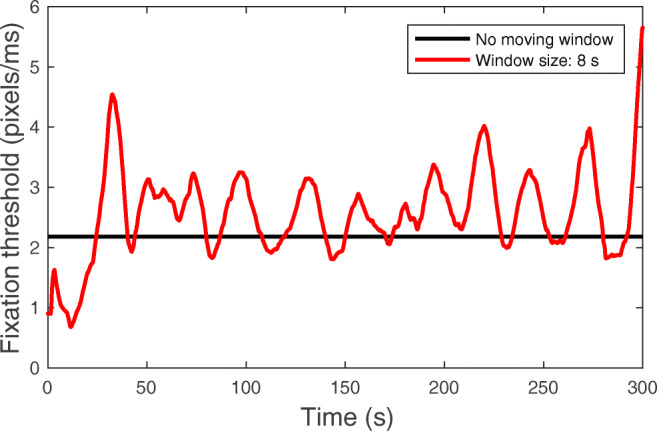
Threshold for fixation classification as a function of time for one example recording. The *red line* depicts the velocity threshold determined using a moving window technique as was used for the data analysis in the present study. The *black line* depicts the velocity threshold determined without using a moving window as has been used in previous work (Hooge & Camps, 2013)

**Fig. 11:**
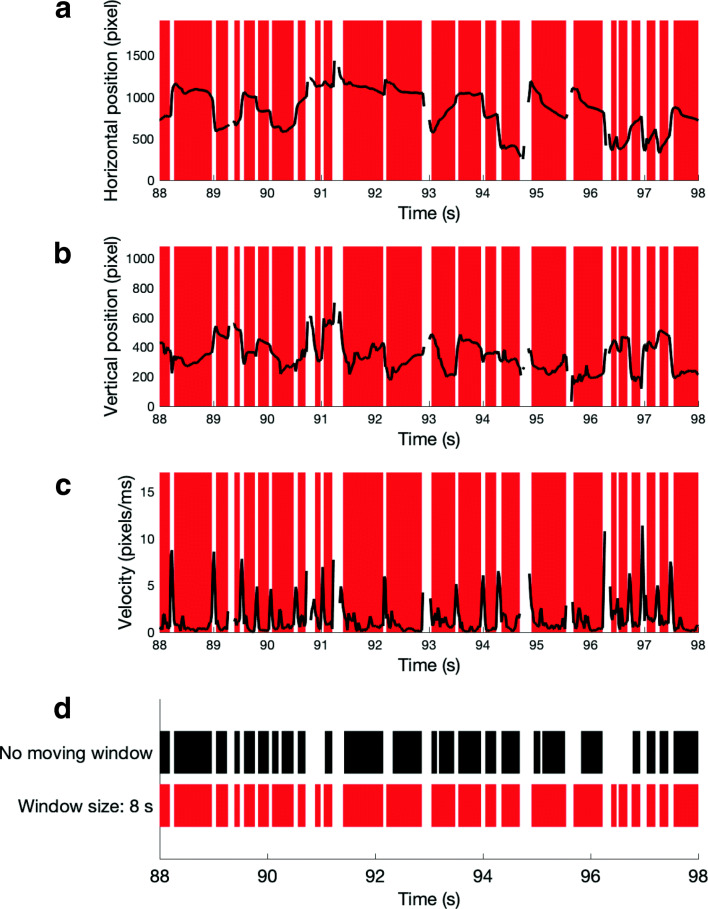
Example eye-tracking data with fixation-classification results for a 10-s episode of one recording. **a** Horizontal gaze position in pixels of the scene camera video. The parts marked in *red* represent the fixations that were classified by our moving window technique. **b** Vertical gaze position in pixels of the scene camera video. The parts marked in *red* represent the fixations that were classified by our moving window technique. **c** Gaze velocity in pixels per ms. The parts marked in *red* represent the fixations that were classified by our moving window technique. **d** Fixations classified with (*marked in red*) and without (*marked in black*) the moving window technique. The fixations classified without the moving window technique were obtained by determining one velocity threshold based on the gaze velocities obtained during the entire recording

## Appendix A: Fixation classification

Figure 10 demonstrates how the threshold for fixation classification depends on whether or not the moving window of 8 s was used to determine the velocity threshold. When no moving window is used for determining the threshold for classifying a sample as a potential fixation sample, the threshold is the same throughout the entire recording. When a moving window is used, the threshold is dependent on the velocities recorded within an 8-s period around the sample. Figure 11 further demonstrates the difference in classified fixations between the two methods. As can be seen in the bottom panel, the fixations classified differ between the two methods. For example at around 90 s, the fixation-classification method without a moving window classifies an additional fixation, whereas additional fixations are classified with the moving window fixation classification at around 96.5 s. On the basis of subjective assessments of the fixation classification, we judged the 8-s moving window to yield the best fixation-classification results overall.

## Footnotes

1 Note that in screen-based eye tracking, fixations and dwells are often distinguished, where one dwell may encompass multiple fixations to the same AOI. Given the speed of the observer relative to the walkers and the environment, multiple fixations to the same person or same place in the environment occurred seldomly.

2 Note that effect sizes are not reported by JASP for the Bayesian repeated-measures ANOVA.

3 Following up on this statement, we checked whether our findings were gender-dependent (we had six male and five female observers). Patterns of gaze behavior in round 1, as well as differences in gaze behavior between round 1 and 2 were very similar for the males and females.

4 https://www.tobiipro.com/product-listing/tobii-pro-glasses-2/#Specificationshttps://www.tobiipro.com/product-listing/tobii-pro-glasses-2/#Specifications, accessed 17-04-2019
